# O02 TRAIL, IP-10, CRP host-protein signature score distinguishes between viral and bacterial infection in sepsis patients

**DOI:** 10.1093/jacamr/dlac052.002

**Published:** 2022-05-31

**Authors:** Jeroen Stas, Cesar A Arias, Richard Bachur, Susanna Esposito, Salim Halabi, Sheldon K Kaplan, Adi Klein, Sergey M Motov, Richard Rothman, Leticia M Ryan, Shachaf Shiber, Tobias Tenenbaum, Alexandra Weissman

**Affiliations:** 1 MeMed, Tirat Carmel, Israel; 2 University of Texas Health Science Center at Houston (UTHealth), 6431 Fannin St, Houston, TX 77030, USA; 3 Houston Methodist Hospital, 6560 Fannin St., Houston, TX 77030, USA; 4 Boston Children’s Hospital, 300 Longwood Ave GL 140, Boston, MA 02115, USA; 5 Pediatric Clinic, Pietro Barilla Children’s Hospital, Department of Medicine and Surgery, University of Parma, Parma, Italy; 6 Carmel Medical Center, Mikhal St. 7, Haifa, 3436212, Israel; 7 Texas Children's Hospital, Feigin Center, 1102 Bates Avenue, Houston, TX 77030, USA; 8 Hillel Yaffe Medical Center, Ha-Shalom St., Hadera 38100, Israel; 9 Maimonides Medical Center, Emergency Medicine 965 48th Street, Brooklyn, NY 11219, USA; 10 Johns Hopkins University, 1800 Orleans Street, Sheikh Zayed Tower, Baltimore, MD 21209, USA; 11 Rabin Medical Center, Zeev Jabotinsky St 39, Petah Tikva, 49100, Israel; 12 Sana Klinikum Lichtenberg, Fanningerstraße 32, 10365 Berlin, Germany; 13 University of Pittsburgh Medical Center, 3550 Terrace Street, Pittsburgh, PA 15261, USA

## Abstract

**Background:**

Sepsis is a life-threatening organ dysfunction syndrome caused by the body's response to infection. Timely and appropriate sepsis management, including appropriate treatment of bacterial infection, improves outcomes. MeMed BV (BV), a test for differentiating between bacterial and viral infection, is based on computational integration of the circulating levels of three proteins (TRAIL, IP-10, CRP). Here we evaluate its ability to differentiate bacterial from viral infection in sepsis patients.

**Methods:**

This was a sub-analysis of sepsis patients recruited prospectively in the Apollo study (NCT04690569). Apollo eligibility required the attending physician's clinical suspicion of acute infection and reported fever. Sepsis was defined as two or more SIRS criteria and a suspected bacterial or viral infection classified by expert adjudication. A bacterial or viral classification required at least 2/3 experts to assign the same aetiology label with confidence ≥90% or all 3 assign with confidence ≥70%. BV was measured using a platform generating a bacterial likelihood score (0–100). Based on pre-defined thresholds, scores 0–34 indicated viral (or other non-bacterial) infection, scores 35 to 65 were equivocal and 66–100 indicated bacterial infection (or coinfection). BV performance was assessed against expert panel classifications.

**Results:**

Seventy-nine out of 1016 eligible Apollo patients had missing heart rate and respiration rate data and a further 136 could not be classified by the experts. Out of the remaining 801 patients, 217 adults with median age of 41.8 years (IQR: 29.2–61) and 149 children with median age of 2.4 years (IQR: 1.4–5.4) had two or more SIRS criteria. A total of 119 patients had at least three SIRS criteria and 39.6% (145/366) of the patients were hospitalized with a median duration of 4 days (IQR: 3–6 days). In the sepsis cohort, 91 patients were classified as bacterial and 275 as viral. BV yielded sensitivity and specificity of 98.8% (95% CI: 93.6%–100%) and 89.7% (95% CI: 85.3%–93.2%) and NPV of 99.6% (95% CI: 97%–99.9%), outperforming PCT [cut-off 0.5 ng/mL; sensitivity 52.8% (95% CI: 42%–63.3%); specificity 86.2% (95% CI: 81.5%–90%); NPV 84.6% (95% CI: 81.5%–87.3%)].

**Conclusions:**

BV accurately distinguished bacterial from viral infection in sepsis patients. This new triage tool has the potential to help with timely identification of bacterial infection, enabling prompt treatment.

